# 4-Hy­droxy­indan-1-one

**DOI:** 10.1107/S1600536812041669

**Published:** 2012-10-10

**Authors:** Che-Wei Chang, Sin-Kai Fang, Ming-Hui Luo, Hsing-Yang Tsai, Kew-Yu Chen

**Affiliations:** a Department of Chemical Engineering, Feng Chia University, 40724 Taichung, Taiwan

## Abstract

The mol­ecule of the title compound, C_9_H_8_O_2_, is essentially planar except for the methyl­ene H atoms [maximum deviation = 0.028 (1) Å]. In the crystal, the mol­ecules are linked by classical O—H⋯O hydrogen bonds and weak C—H⋯O inter­actions into chains along [110] and [1-10].

## Related literature

For the preparation of the title compound, see: Gerasov *et al.* (2011 ▶). For applications of indanone derivatives, see: Tang *et al.* (2011 ▶); Borbone *et al.* (2011 ▶); Borge *et al.* (2010 ▶); Cai *et al.* (2005 ▶); Cui *et al.* (2009 ▶); Fu & Wang (2008 ▶); Li *et al.* (2009 ▶); Sousa *et al.* (2011 ▶); Yu *et al.* (2011 ▶). For related structures, see: Ali *et al.* (2010 ▶); Chen *et al.* (2011*a* ▶,*b* ▶). For graph-set theory, see: Bernstein *et al.* (1995 ▶).
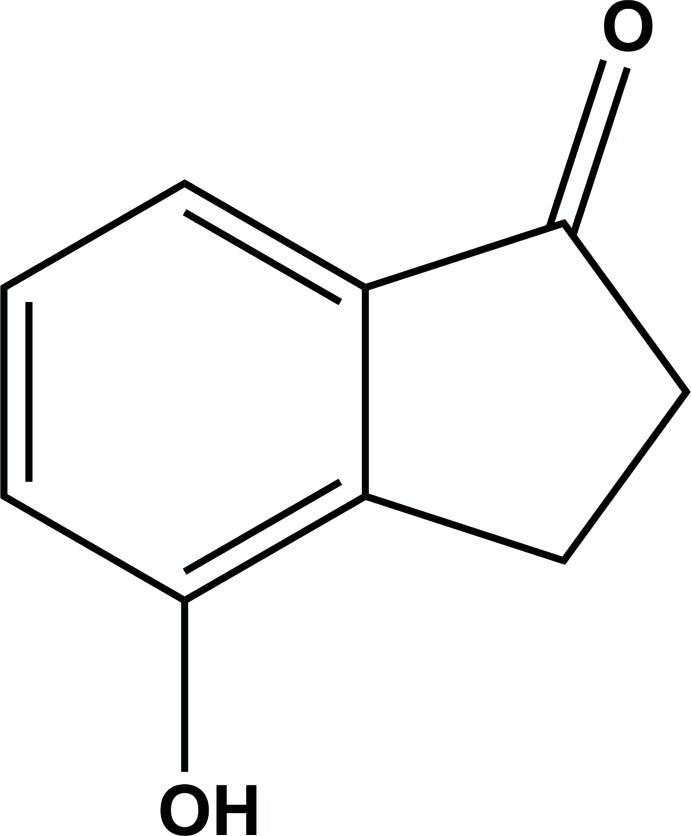


## Experimental

### 

#### Crystal data

C_9_H_8_O_2_
*M* *_r_* = 148.15
Monoclinic, 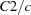

*a* = 13.5890 (6) Å
*b* = 8.6160 (3) Å
*c* = 13.9435 (6) Å
β = 116.738 (6)°
*V* = 1457.98 (13) Å^3^
*Z* = 8
Mo *K*α radiation
μ = 0.10 mm^−1^
*T* = 297 K
0.63 × 0.60 × 0.38 mm

#### Data collection

Bruker SMART CCD detector diffractometer
6305 measured reflections
1794 independent reflections
1289 reflections with *I* > 2σ(*I*)
*R*_int_ = 0.018

#### Refinement

*R*[*F* ^2^ > 2σ(*F* ^2^)] = 0.040
*wR*(*F* ^2^) = 0.115
*S* = 1.06
1794 reflections
104 parameters
H atoms treated by a mixture of independent and constrained refinement
Δρ_max_ = 0.21 e Å^−3^
Δρ_min_ = −0.15 e Å^−3^

### 

Data collection: *SMART* (Bruker, 2005 ▶); cell refinement: *SAINT* (Bruker, 2005 ▶); data reduction: *SAINT*; program(s) used to solve structure: *SHELXS97* (Sheldrick, 2008 ▶); program(s) used to refine structure: *SHELXL97* (Sheldrick, 2008 ▶); molecular graphics: *ORTEP-3 for Windows* (Farrugia, 1997 ▶); software used to prepare material for publication: *WinGX* publication routines (Farrugia, 1999 ▶).

## Supplementary Material

Crystal structure: contains datablock(s) global, I. DOI: 10.1107/S1600536812041669/xu5628sup1.cif

Structure factors: contains datablock(s) I. DOI: 10.1107/S1600536812041669/xu5628Isup2.hkl

Additional supplementary materials:  crystallographic information; 3D view; checkCIF report

## Figures and Tables

**Table 1:** Hydrogen-bond geometry (Å, °)

*D*—H⋯*A*	*D*—H	H⋯*A*	*D*⋯*A*	*D*—H⋯*A*
O1—H1*A*⋯O2^i^	0.94 (2)	1.75 (2)	2.6918 (18)	175 (2)
C2—H2*A*⋯O2^i^	0.93	2.59	3.255 (2)	129

Symmetry code: (i) 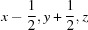.

**Fig. 1.**
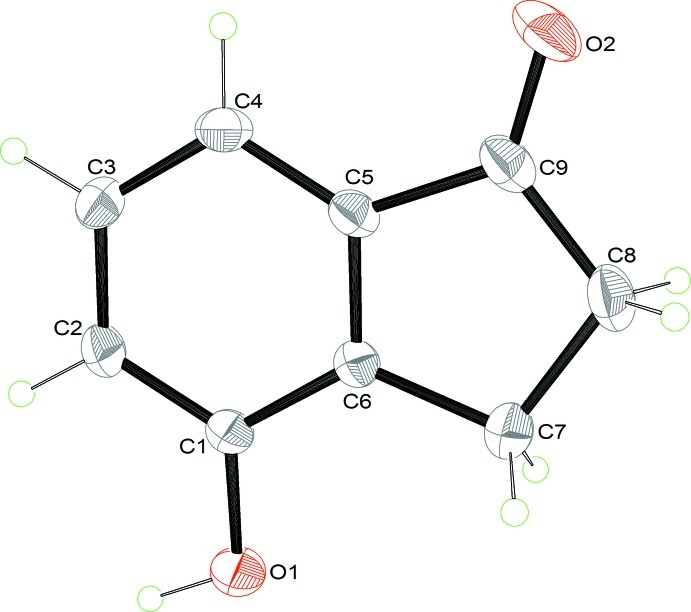
The molecular structure of the title compound, showing 50% probability displacement ellipsoids.

**Fig. 2.**
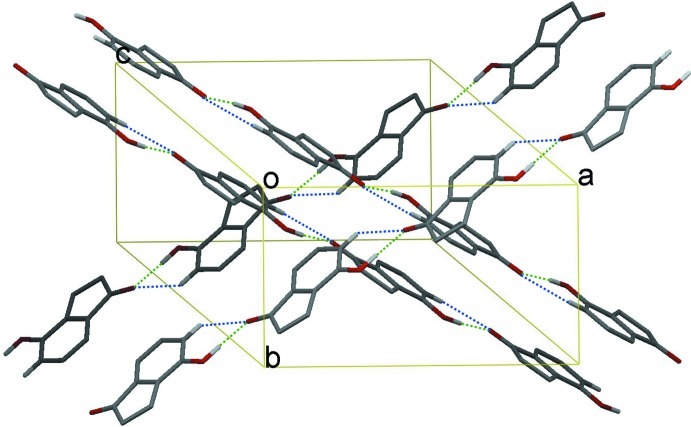
A section of the crystal packing of the title compound, viewed along the c axis. Green and blue dashed lines denote the intermolecular O1—H1A···O2 and C2—H2A···O2 hydrogen bonds, respectively. For clarity, hydrogen atoms not involved in hydrogen bonding have been omitted.

## supplementary crystallographic information

### Comment

Indanone derivatives are some of the most widely used organic compounds (Tang *et al.*, 2011). Indanone derivatives are used as dyes and pigments (Cui *et al.*, 2009; Li *et al.*, 2009), intermediates in organic synthesis (Borbone *et al.*, 2011; Borge *et al.*, 2010; Fu & Wang, 2008; Yu *et al.*, 2011), and exhibit a wide variety of biological activities (Sousa *et al.*, 2011). In addition, 1-indanones were important precursors in the regiospecific synthesis of 2-fluoro-1-naphthols (Cai *et al.*, 2005).

The molecular structure of the title compound is shown in Figure 1. The molecule is essentially planar (the maximum deviation = 0.028 (1) Å), which is consistent with previous studies (Ali *et al.*, 2010; Chen *et al.*, 2011*a*,*b*). In the crystal (Fig. 2), molecules are linked by intermolecular O—H···O and C—H···O hydrogen bonds (Table 1) to form an infinite one-dimensional chain along and [1 1 0 and ][1 -1 0], generating two different kinds of C(7) motifs (Bernstein *et al.*, 1995).

### Experimental

The title compound was synthesized by the hydrolation of 4-benzoyloxy-1-indanone with sodium hydroxide (Gerasov *et al.*, 2011). Colorless parallelepiped -shaped crystals suitable for the crystallographic studies reported here were isolated over a period of five weeks by slow evaporation from a ethyl acetate solution.

### Refinement

H atoms bonded to O and C atoms were located in a difference electron density map. The hydroxy H atom and the C_sp3_ H atoms were freely refined, and the C_sp2_ H atoms repositioned geometrically and refined using a riding model, [C—H = 0.93 Å and *U*_iso_(H) = 1.2*U*_eq_(C)].

### Crystal data

**Table:**

C_9_H_8_O_2_	*F*(000) = 624
*M**_r_* = 148.15	*D*_x_ = 1.350 Mg m^−^^3^
Monoclinic, *C*2/*c*	Mo *K*α radiation, λ = 0.71073 Å
Hall symbol: -C 2yc	Cell parameters from 3232 reflections
*a* = 13.5890 (6) Å	θ = 2.8–29.2°
*b* = 8.6160 (3) Å	µ = 0.10 mm^−^^1^
*c* = 13.9435 (6) Å	*T* = 297 K
β = 116.738 (6)°	Parallelepiped, colorless
*V* = 1457.98 (13) Å^3^	0.63 × 0.60 × 0.38 mm
*Z* = 8	

### Data collection

**Table:**

Bruker SMART CCD detector diffractometer	1289 reflections with *I* > 2σ(*I*)
Radiation source: fine-focus sealed tube	*R*_int_ = 0.018
Graphite monochromator	θ_max_ = 29.2°, θ_min_ = 2.9°
ω scans	*h* = −18→18
6305 measured reflections	*k* = −11→11
1794 independent reflections	*l* = −19→18

### Refinement

**Table:**

Refinement on *F*^2^	Primary atom site location: structure-invariant direct methods
Least-squares matrix: full	Secondary atom site location: difference Fourier map
*R*[*F*^2^ > 2σ(*F*^2^)] = 0.040	Hydrogen site location: inferred from neighbouring sites
*wR*(*F*^2^) = 0.115	H atoms treated by a mixture of independent and constrained refinement
*S* = 1.06	*w* = 1/[σ^2^(*F*_o_^2^) + (0.0704*P*)^2^] where *P* = (*F*_o_^2^ + 2*F*_c_^2^)/3
1794 reflections	(Δ/σ)_max_ = 0.001
104 parameters	Δρ_max_ = 0.21 e Å^−^^3^
0 restraints	Δρ_min_ = −0.15 e Å^−^^3^

### Special details

**Table:**

Geometry. All e.s.d.'s (except the e.s.d. in the dihedral angle between two l.s. planes) are estimated using the full covariance matrix. The cell e.s.d.'s are taken into account individually in the estimation of e.s.d.'s in distances, angles and torsion angles; correlations between e.s.d.'s in cell parameters are only used when they are defined by crystal symmetry. An approximate (isotropic) treatment of cell e.s.d.'s is used for estimating e.s.d.'s involving l.s. planes.
Refinement. Refinement of *F*^2^ against ALL reflections. The weighted *R*-factor *wR* and goodness of fit *S* are based on *F*^2^, conventional *R*-factors *R* are based on *F*, with *F* set to zero for negative *F*^2^. The threshold expression of *F*^2^ > σ(*F*^2^) is used only for calculating *R*-factors(gt) *etc*. and is not relevant to the choice of reflections for refinement. *R*-factors based on *F*^2^ are statistically about twice as large as those based on *F*, and *R*- factors based on ALL data will be even larger.

### Fractional atomic coordinates and isotropic or equivalent isotropic displacement parameters (Å^2^)

**Table:**

	*x*	*y*	*z*	*U*_iso_*/*U*_eq_	
O1	0.21183 (9)	0.05958 (13)	0.03489 (8)	0.0607 (3)	
H1A	0.1454 (17)	0.116 (3)	0.0012 (17)	0.101 (6)*	
O2	0.51759 (9)	−0.29276 (13)	−0.06930 (10)	0.0714 (4)	
C1	0.24288 (9)	0.01208 (14)	−0.04004 (9)	0.0373 (3)	
C2	0.18722 (10)	0.05169 (14)	−0.14805 (10)	0.0405 (3)	
H2A	0.1252	0.1147	−0.1716	0.049*	
C3	0.22258 (10)	−0.00108 (14)	−0.22117 (9)	0.0414 (3)	
H3A	0.1839	0.0273	−0.2930	0.050*	
C4	0.31337 (10)	−0.09427 (14)	−0.18965 (10)	0.0404 (3)	
H4A	0.3372	−0.1300	−0.2386	0.049*	
C5	0.36864 (9)	−0.13348 (13)	−0.08148 (9)	0.0350 (3)	
C6	0.33542 (9)	−0.08162 (13)	−0.00686 (9)	0.0341 (3)	
C7	0.40960 (11)	−0.13665 (16)	0.10481 (10)	0.0470 (3)	
H7A	0.3686	−0.1952	0.1343	0.056*	
H7B	0.4457	−0.0499	0.1519	0.056*	
C8	0.49369 (11)	−0.24025 (16)	0.09019 (12)	0.0499 (4)	
H8A	0.5681	−0.2036	0.1346	0.060*	
H8B	0.4879	−0.3465	0.1100	0.060*	
C9	0.46657 (10)	−0.23041 (14)	−0.02658 (11)	0.0445 (3)	

### Atomic displacement parameters (Å^2^)

**Table:**

	*U*^11^	*U*^22^	*U*^33^	*U*^12^	*U*^13^	*U*^23^
O1	0.0637 (7)	0.0832 (8)	0.0452 (6)	0.0363 (6)	0.0333 (5)	0.0071 (5)
O2	0.0529 (6)	0.0864 (8)	0.0794 (8)	0.0316 (6)	0.0337 (6)	−0.0066 (6)
C1	0.0363 (6)	0.0424 (6)	0.0357 (6)	0.0098 (5)	0.0186 (5)	0.0002 (5)
C2	0.0350 (6)	0.0421 (7)	0.0416 (7)	0.0121 (5)	0.0149 (5)	0.0066 (5)
C3	0.0460 (7)	0.0437 (7)	0.0305 (6)	0.0028 (5)	0.0135 (5)	0.0024 (5)
C4	0.0442 (7)	0.0446 (7)	0.0373 (6)	0.0016 (5)	0.0226 (6)	−0.0067 (5)
C5	0.0305 (6)	0.0354 (6)	0.0398 (6)	0.0029 (5)	0.0162 (5)	−0.0040 (4)
C6	0.0315 (6)	0.0365 (6)	0.0327 (6)	0.0047 (5)	0.0130 (5)	−0.0003 (4)
C7	0.0428 (7)	0.0557 (8)	0.0357 (7)	0.0110 (6)	0.0118 (6)	0.0040 (5)
C8	0.0353 (7)	0.0491 (7)	0.0532 (8)	0.0116 (6)	0.0091 (6)	0.0054 (6)
C9	0.0329 (6)	0.0425 (7)	0.0559 (8)	0.0069 (5)	0.0181 (6)	−0.0051 (6)

### Geometric parameters (Å, º)

**Table:**

O1—C1	1.3542 (13)	C4—H4A	0.9300
O1—H1A	0.94 (2)	C5—C6	1.3814 (15)
O2—C9	1.2229 (14)	C5—C9	1.4622 (16)
C1—C6	1.3869 (15)	C6—C7	1.5011 (16)
C1—C2	1.3899 (17)	C7—C8	1.5341 (18)
C2—C3	1.3848 (16)	C7—H7A	0.9700
C2—H2A	0.9300	C7—H7B	0.9700
C3—C4	1.3683 (16)	C8—C9	1.501 (2)
C3—H3A	0.9300	C8—H8A	0.9700
C4—C5	1.3906 (16)	C8—H8B	0.9700
			
C1—O1—H1A	109.4 (12)	C1—C6—C7	127.94 (10)
O1—C1—C6	117.97 (10)	C5—C6—C7	112.62 (10)
O1—C1—C2	123.73 (11)	C6—C7—C8	103.81 (10)
C6—C1—C2	118.30 (10)	C6—C7—H7A	111.0
C1—C2—C3	121.15 (11)	C8—C7—H7A	111.0
C1—C2—H2A	119.4	C6—C7—H7B	111.0
C3—C2—H2A	119.4	C8—C7—H7B	111.0
C4—C3—C2	121.17 (11)	H7A—C7—H7B	109.0
C4—C3—H3A	119.4	C9—C8—C7	106.09 (10)
C2—C3—H3A	119.4	C9—C8—H8A	110.5
C3—C4—C5	117.38 (10)	C7—C8—H8A	110.5
C3—C4—H4A	121.3	C9—C8—H8B	110.5
C5—C4—H4A	121.3	C7—C8—H8B	110.5
C4—C5—C6	122.57 (10)	H8A—C8—H8B	108.7
C4—C5—C9	128.81 (10)	O2—C9—C5	125.30 (13)
C6—C5—C9	108.63 (10)	O2—C9—C8	125.95 (12)
C1—C6—C5	119.44 (10)	C5—C9—C8	108.75 (10)

### Hydrogen-bond geometry (Å, º)

**Table:**

*D*—H···*A*	*D*—H	H···*A*	*D*···*A*	*D*—H···*A*
O1—H1*A*···O2^i^	0.94 (2)	1.75 (2)	2.6918 (18)	175 (2)
C2—H2*A*···O2^i^	0.93	2.59	3.255 (2)	129

Symmetry code: (i) *x*−1/2, *y*+1/2, *z*.

## References

[bb1] Ali, M. A., Ismail, R., Tan, S. C., Yeap, C. S. & Fun, H.-K. (2010). *Acta Cryst.* E**66**, o2864. 10.1107/S1600536810040869 PMC3008978 21589046

[bb2] Bernstein, J., Davis, R. E., Shimoni, L. & Chang, N.-L. (1995). *Angew. Chem. Int. Ed. Engl.* **34**, 1555–1573.

[bb3] Borbone, F., Carella, A., Ricciotti, L., Tuzi, A., Roviello, A. & Barsella, A. (2011). *Dyes Pigm.* **88**, 290–295.

[bb4] Borge, J., Cadierno, V., Díez, J., García-Garrido, S. E. & Gimeno, J. (2010). *Dyes Pigm.* **87**, 209–217.

[bb5] Bruker (2005). *SMART* and *SAINT* Bruker AXS Inc., Madison, Wisconsin, USA.

[bb6] Cai, X., Wu, K. & Dolbier, W. R. Jr (2005). *J. Fluor. Chem.* **126**, 479–482.

[bb7] Chen, K.-Y., Fang, T.-C. & Chang, M.-J. (2011*a*). *Acta Cryst.* E**67**, o992. 10.1107/S1600536811010956 PMC3099973 21754249

[bb8] Chen, K.-Y., Wen, Y.-S., Fang, T.-C., Chang, Y.-J. & Chang, M.-J. (2011*b*). *Acta Cryst.* E**67**, o927. 10.1107/S1600536811009718 PMC3099748 21754197

[bb9] Cui, Y., Ren, H., Yu, J., Wang, Z. & Qian, G. (2009). *Dyes Pigm.* **81**, 53–57.

[bb10] Farrugia, L. J. (1997). *J. Appl. Cryst.* **30**, 565.

[bb11] Farrugia, L. J. (1999). *J. Appl. Cryst.* **32**, 837–838.

[bb12] Fu, T.-L. & Wang, I.-J. (2008). *Dyes Pigm.* **76**, 590–595.

[bb13] Gerasov, A. O., Zyabrev, K. V., Shandura, M. P. & Kovtun, Y. P. (2011). *Dyes Pigm.* **89**, 76–85.

[bb14] Li, X., Kim, S. H. & Son, Y. A. (2009). *Dyes Pigm.* **82**, 293–298.

[bb15] Sheldrick, G. M. (2008). *Acta Cryst.* A**64**, 112–122. 10.1107/S0108767307043930 18156677

[bb16] Sousa, C. M., Berthet, J., Delbaere, S. & Coelho, P. J. (2011). *Dyes Pigm.* **92**, 537–541.

[bb17] Tang, K.-C., Chang, M.-J., Lin, T.-Y., Pan, H.-A., Fang, T.-C., Chen, K.-Y., Hung, W.-Y., Hsu, Y.-H. & Chou, P.-T. (2011). *J. Am. Chem. Soc.* **133**, 17738–17745. 10.1021/ja2062693 21957929

[bb18] Yu, S.-B., Liu, H.-M., Luo, Y. & Lu, W. (2011). *Chin. Chem. Lett.* **22**, 264–267.

